# Examining the job burnout of Chinese hospitality management students in internships *via* the transactional model

**DOI:** 10.3389/fpsyg.2022.973493

**Published:** 2022-11-17

**Authors:** Xiao L. Yin, Yan L. Yang, Hyung J. Kim, Yan Zhang

**Affiliations:** ^1^School of Hotel Management, Qingdao Vocational and Technical College of Hotel Management, Qingdao, China; ^2^College of International Education, Ludong University, Yantai, China; ^3^School of Business, Chungnam National University, Daejeon, South Korea

**Keywords:** contextual factors, hospitality management intern, job burnout, personal factors, transactional model

## Abstract

The ongoing COVID-19 pandemic has increased the psychological burden on employees in hotels, which is not conducive to the development of the hospitality industry. Based on a survey of 379 hotel interns from higher vocational colleges in China, this study empirically analyzed the status quo of job burnout in future hotel employees and its influencing factors. The results showed that interns’ job burnout and reduced personal accomplishment were at a medium level. Secondly, according to the transaction model, this study classified the antecedents of job burnout into two categories: personal factors and contextual factors. The results showed that personal factors such as attitude and self-efficacy, and contextual factors such as perceived co-worker support and job satisfaction all had a negative effect on job burnout. However, the influence of ability and perceived supervisor support on job burnout was not significant. This study also investigated the influencing factors of each sub-dimension of job burnout. Self-efficacy, attitude and job satisfaction all had a negative influence on the three sub-dimensions. Ability and perceived co-worker support only had a negative impact on reduced personal accomplishment. There was no statistical correlation between perceived supervisor support and the three sub-dimensions. The results of this study will lay a theoretical foundation so that higher vocational colleges can better organize and implement internships, and hotels can recruit energetic future employees.

## Introduction

Job burnout is a psychological syndrome that refers to a continuous emotional or extreme response shown as an individual’s inability to effectively cope with various pressures at work and interpersonal stressors (Li and Shi, 2003). It is not a disease on its own, but can put the individual into a risk state known as “sub-healthy.” Job burnout is closely related to individual health and turnover intention (Schaufeli and Bakker, 2004). Long-term burnout can cause headaches, depression, anxiety, etc., and can also affect an individual’s work, such as low work efficiency, decreased service quality and job satisfaction, decreased organizational commitment and increased absenteeism or turnover intention. This leads to high production losses and increased medical and health care costs (Von Känel et al., 2016; Yetgin and Benligiray, 2019).

Job burnout is shown in three forms: emotional exhaustion (EE), depersonalization (DP), and reduced personal accomplishment (PA). Emotional exhaustion is the core dimension of burnout. It refers to individuals who think that their energy or emotional resources are exhausted, feel tired and stressed, and lack motivation to work. Depersonalization is the interpersonal dimension, which means that individuals intentionally separate from work or others, and passively complete the assigned tasks. Reduced personal accomplishment is the self-evaluation dimension of burnout, which refers to a negative evaluation held by individuals regarding themselves. That is, individuals think they are not competent for the current job, or doubt that their activities make a contribution (Lizano and Mor Barak, 2015).

Job burnout widely exists in professionals who provide social and human services. Therefore, relevant academic research has focused on medical staff (Hsieh et al., 2021; Wei et al., 2021), teachers (Chen et al., 2020; Lu et al., 2021; Tian et al., 2021), tour guides (Li and Chen, 2012; Yetgin and Benligiray, 2019) and those in other industries, but there is a lack of systematic research on hotel interns.

Hotel work is a special occupation, known for its labor intensive and intense interpersonal interaction. Therefore, it requires employees to invest a lot of energy, and also demands strong sustainability and has high expectations in terms of service goals. Employees engaged in the hotel industry have to face all kinds of guests every day, with a heavy workload, high intensity and mechanized repetitive work. Moreover, the employees must respond with minimal error rates to serve customers who have different needs. As a result, the high rate of job burnout in the hospitality industry has been increasing (Harjanti and Todani, 2019). In 2020, the sudden outbreak of COVID-19 hit the hotel industry hard, followed by the epidemic prevention protocols, a wave of closures, a wave of resignations and so on. This increased the workload and psychological pressure of hotel staff. Early work on industries related to tourism found that role conflict and poor communication can lead to job burnout. Interns who have just entered the hotel from school are in the role transition period, have no in-depth understanding of work and lack necessary experience. Compared with regular employees, they have a weak sense of belonging to the enterprise and strong work pressure. If there is no suitable way to relieve or pour out this pressure or emotion, it is very easy for burnout to occur. Once job burnout occurs, interns will inevitably lose their motivation to work and confidence in their career prospects, and it may even affect their future career planning. Therefore, empirical research on job burnout in hotel interns under the current epidemic prevention measures is urgent and important.

## Literature review and development of hypotheses

### Job burnout

Job burnout was first proposed by Freudenberger, and scholars believe that this symptom is most likely to occur in the helping industries. Later, Maslach and other scholars extended the concept of job burnout into three dimensions-emotional exhaustion, depersonalization and reduced personal accomplishment-and developed the Maslach Burnout Inventory General Survey (MBI-GS). This interpretation has been recognized as a classic and is constantly cited. The scale has also been verified and used in many countries and regions, and has been subdivided into the Maslach Burnout Inventory Educator Survey (MBI-ES), the Maslach Burnout Inventory Nurse Survey (MBI-NS) and the Maslach Burnout Inventory Student Survey (MBI-SS). The Chinese scholars Li and Shi (2003) translated and revised the Chinese burnout inventory general survey according to the MBI-GS.

Since the 1970s, scholars have carried out many studies on the definition, measurement, antecedents and outcome variables of job burnout. Regarding the antecedents, different scholars have continually discussed these in different professional fields. Li and Shi (2003) suggested that enterprises should pay more attention to the job burnout of young employees after analyzing job burnout from the perspective of demographic and organizational justice. He (2011) concluded from the investigations that students’ academic performance was significantly negatively correlated with burnout and its three sub-dimensions. Li and Chen (2012) believed that role conflict, job control, and organizational or social support were the main reasons for emotional exhaustion in travel agency managers. Chan et al. (2015) studied the relationships among job burnout, job satisfaction and turnover intention from the perspectives of demography, shift work and job position. The results showed that female staff in the hotel suffered more stress than male staff, with shift workers more likely to suffer burnout. Von Känel et al. (2016) believed that demographic and psychological factors, such as age, socioeconomic status, social support and sleep problems, etc., were related to burnout. Females scored higher in terms of emotional exhaustion, whereas males scored higher in terms of depersonalization. Prentice and Thaichon (2019) believed that job performance and occupational commitment negatively affect job burnout. Lu et al. (2021) and Chen et al. (2020) found that teachers’ professional identity and job satisfaction had a significant negative influence on job burnout. Du et al. (2021) believed that age, length of work and education level were the main factors affecting job burnout in hospital managers. The shorter the working life, the more likely employees were to long for escape and the greater the reduction in personal accomplishment.

Students leave school and enter hotels for internships. They want to comprehensively use the basic knowledge and skills that they have learned to gain a preliminary perceptual understanding of work, develop the correct professional attitude and take a positive first step on their career paths. An internship is an important early career stage for students, and it is difficult to establish a new role, which can easily lead to burnout. The research of Goddard and Goddard (2006) confirmed that there is a strong correlation between turnover intention and job burnout at the beginning of the career. According to China’s Hotel Human Resources Survey Report (2018), the turnover rate of newcomers in the hospitality industry within 3 years is high, accounting for more than 90% of the total number of leavers. Corresponding to the high turnover rate is the increasing shortage of employees in various departments of the hotel. Existing research on job burnout during internships are quite limited, which may be attributed to the fact that burnout is caused by long-term accumulation of stress. However, there is evidence that burnout, though a gradual process, has its roots in pre-employment training or internships (Gavish and Friedman, 2010).

*H1*: The hospitality Management Students will suffer from job burnout during their internship.

### Theoretical foundations

The existing literature has identified multiple sources of stress during an internship, but studies have rarely combined contextual and personal factors. Cano-García et al. (2005) confirmed that the combination of personal (such as personality, perceived self-efficacy, etc.) and contextual variables (such as sources of perceived stress, job satisfaction, perceived social support, etc.) can significantly predict job burnout. Chan (2003) called for an in-depth understanding of the personal and contextual factors that may trigger job burnout, as well as coping strategies in the early phase of a career. Based on the Transactional Model of job-stress and burnout (Kokkinos and Stavropoulos, 2014), which extensively studies job stress and burnout, this study analyzed the burnout of interns. The model indicates that the interaction between the personal and contextual variables determine whether the situation puts pressure on the individual. Therefore, burnout can be interpreted as a result of the interaction between triggering contextual and personal variables.

#### Personal variables and job burnout

Any kind of activity, including an internship, requires an individual to possess certain abilities, that is, a combination of knowledge and skills. Ability is the degree to which an individual possesses the necessary resources to make the desired result a reality. It is a capability or power that directly affects the efficiency of the activity. The higher the individual’s ability to process information, the more motivated she/he will be. Interns can only be competent to work and achieve the desired performance when they fully possess the required ability. Ability is one of the psychological characteristics of personality, and there are obvious differences between individuals. Therefore, interns with stronger ability are less likely to experience job burnout. He (2011) verified that academic achievement was significantly negatively correlated with job burnout and its sub-dimensions.

The theory of planned behavior states that an individual’s behavioral intention depends on attitudes and subjective norms. “An attitude is an overall evaluation that expresses how much we like or dislike an object, issue, person, or action” (Hoyer and MacInnis, 2013). Attitudes can be learned and, once formed, become a part of one’s personality, which persist and are resistant to change. Pinna et al. (2020) defined an employee’s attitude as an emotional state based on their experience in a social environment. Attitude is important because it can guide individual thoughts, affect feelings and behavior. Attitude consists of significant beliefs, including perception of possibilities and judgments about the consequences of actions. There is a strong association between attitude and job burnout (Taylor et al., 2019). The more negative the attitude, the more an individual appeared to suffer from emotional exhaustion and depersonalization, and score lower in terms of personal accomplishment (Consiglio et al., 2013).

As a central component of social cognitive theory, self-efficacy refers to an individual’s confidence about whether they can use the skills or abilities that they possess to complete a task or overcome difficulties. It is an individual’s belief or judgment of their ability to successfully execute an action in a specific environment (Aloe et al., 2014; Burger and Samuel, 2017; Huang et al., 2021). Social cognitive theory believes that perceived self-efficacy, as a cognitive determinant of behavior, will affect the individual’s selection of coping strategies in the face of challenging demands (Tu and Zhang, 2015; Yan et al., 2021).

In the job demand–resource model, self-efficacy is considered to be one of the three components of personal resources that contribute to positive human behaviors. Individuals with high self-efficacy will show greater perseverance when they are in a stressful situation, tend to actively solve problems, work hard to improve a disadvantageous situation and generate inner satisfaction from their work. On the contrary, individuals with low self-efficacy are more likely to give up when facing difficulties (Liu et al., 2021). Self-efficacy has been proven to be an important personal resource in an organization, and baseline stress will be neutralized by an individual’s self-efficacy (Burger and Samuel, 2017). Researchers are increasingly using self-efficacy to study job burnout and explore the role of self-efficacy in the formation of job burnout, and have even defined job burnout as “a crisis of self-efficacy” (Consiglio et al., 2013; Yu et al., 2015). Studies have confirmed that there is a significant relationship between self-efficacy and the three dimensions of burnout, and a lower level of self-efficacy may lead to a higher level of burnout (Aloe et al., 2014).

*H2_1*: The ability of hotel interns is significantly negatively correlated with job burnout and its sub-dimensions.

*H2_2*: Attitude toward the internship is significantly negatively correlated with job burnout and its sub-dimensions.

*H2_3*: Self-efficacy is significantly negatively correlated with job burnout and its sub-dimensions.

#### Contextual variables and job burnout

Burnout is not just related to individual, but also to work-related social or environmental issues (Harjanti and Todani, 2019). Most researchers agreed that burnout is best understood in terms of situational stressors (Hu and Cheng, 2010). Support is generally regarded as an important resource to help individuals cope with and adjust to work stress (Duan and Xie, 2015; Blomberg and Rosander, 2020). The support from the workplace is called organizational support. In the hospitality industry, supervisor and co-worker support represent the two main sources of organizational support for employees. Organizational support injects positive emotional resources, help and recognition into the work environment of employees, which is positively correlated, to some extent, with decreased psychological distress (Kokkinos and Stavropoulos, 2014). In the workplace, perceived supervisor support refers to the degree of happiness from being appreciated and valued by the supervisor. The supervisors mainly support employees by providing constructive suggestions and help them fulfill their job responsibilities. For example, an employee who is authorized to make decisions about refunds and upgrade rooms for free can handle hotel emergencies more effectively than an employee who is waiting for instructions from the supervisor, and they find it easier to achieve satisfaction. Perceived co-worker support refers to an individual’s degree of confidence that her/his colleagues will assist them in performing their duties by providing mission-related information and care. A supportive group of colleagues can create a good atmosphere where employees can express happiness and sympathy, grievances and worries in an equal and free environment. The common point of support by supervisors and co-workers is that it can satisfy employees’ need for respect and their sense of belonging, and enhance their sense of workplace comfort (Zhu et al., 2019). When employees perceive a higher level of support from their supervisors or co-workers, this will increase their sense of psychological security and control, thereby buffering the feelings of stress caused by high work demands. There-fore, organizational support is known as the buffer between stressors and stress feedback, and is an important yet independent predictor of job burnout (Duan and Xie, 2015; Kilroy et al., 2021). Resource theories, such as the job demand–resource model and the resource conservation theory, have all pointed out that perceived supervisor support and perceived co-worker support can help reduce job burnout.

On the other hand, job satisfaction is associated with job burnout. Job satisfaction is described as an emotional state caused by an individual’s work experience. In an internship, job satisfaction mainly refers to the overall feelings and opinions of interns regarding their work, occupation, working conditions and status (McAllister et al., 2017). Job satisfaction is regarded as a job resource that has a powerful and farreaching influence on people in the workplace, because it can increase career identity and is related to a positive work attitude and good job performance (Zhai et al., 2013). Studies have shown that job satisfaction is one of the important factors influencing job burnout. Individuals with high job satisfaction have a stronger career identity and more psychological resources to use when coping with negative experiences (such as burnout). However, lower job satisfaction results in low morale and a lack of enthusiasm, which hinders the full development of career identity (Chen et al., 2020; Lu et al., 2021). Individuals’ dissatisfaction with work content, environment and salary will lead to burnout (Lu and Gursoy, 2016). Wu et al. (2009) found that teachers’ job satisfaction had a significant impact on job burnout. The level of job burnout of less satisfied teachers was significantly higher than that of highly satisfied teachers. This study will explore whether this result is applicable to interns.

*H2_4*: Perceived supervisor support is significantly negatively correlated with job burnout and its sub-dimensions.

*H2_5*: Perceived co-worker support is significantly negatively correlated with job burnout and its sub-dimensions.

*H2_6*: Job satisfaction is significantly negatively correlated with job burnout and its sub-dimensions.

### Control variables

Shift work, workload and length of adaptation also have an impact on job burnout (Chan et al., 2015; Hsieh et al., 2021). However, in accordance with the relevant laws and regulations, hotels generally do not arrange night shifts or overtime work when assigning tasks to interns, unless interns voluntarily ask for it. This study controls for the above three variables.

**Figure 1:**
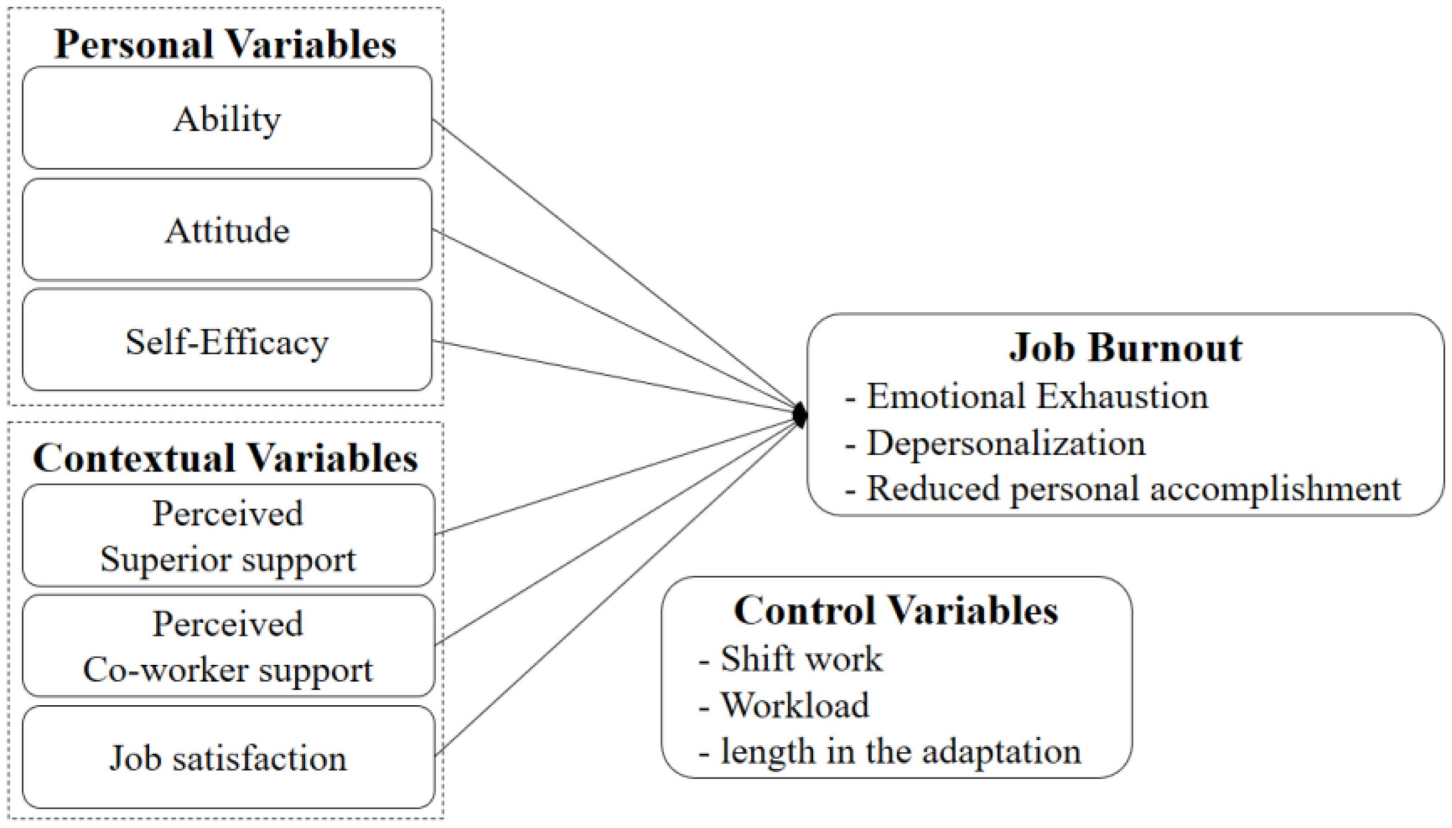
Conceptual model.

The model constructed in this study is shown in Figure 1.

## Research object and design

### Research objects and survey

When students finished their internships in September 2021, the survey team distributed questionnaires online and as hard copy to more than 60 hotels in 14 cities, including Beijing and Shanghai. The specially trained investigator first briefly introduced the purpose of the survey and the method of answering to the interns, emphasizing the principle of voluntary participation. It was made clear that the questionnaire would only be used for academic research and would be kept strictly confidential. If interns felt it was an inconvenience, they could choose to withdraw at any time during the investigation process. In total, 442 questionnaires were issued in this survey, and invalid questionnaires such as incomplete answers were deleted. Finally, 379 questionnaires were obtained, with an effective recovery rate of 85.7%. A t-test was performed after the data had been coded, and no significant statistical difference was found between the data collected online and as hardcopy, indicating that the data could be combined for analysis.

### Research tools

In the model of this study, the measurement items of all variables were based on scales that have been verified in previous literature. The Job Burnout Scale used the instrument of Li and Shi (2003) based on the Maslach Burnout Inventory General Survey (MBI-GS), which was revised to suit the characteristics of Chinese people. The scale consists of 15 items, divided into three dimensions: emotional exhaustion (5 items, e.g., I feel emotionally drained by my internship), depersonalization (4 items, e.g., Since internship, I have gradually lost interest in my work) and reduced personal accomplishment (6 items, e.g., I feel able to complete the work in my internship). A 7-point Likert scale was used, in which 0 means “never” and 6 means “every day.” Among the items, reduced personal accomplishment was reverse-scored and scores were reassigned during the actual analysis. The higher the score of each sub-dimension, the higher the degree of burnout. For each scale, 1–3 was classified as mild, 3–5 was classified as moderate and scores greater than 5 were classified as severe burnout. The comprehensive score of job burnout was calculated by the formula by Burnout Risk Index-original (Brix-O) based on Kalimo et al. (2003), Ahola et al. (2005), and Von Känel et al. (2016).


$$\begin{array}{l} S_{job\;burnout}=0.4\times S_{Emotionalexhaustion}\\ \;\;\;+0.3\times S_{Depersonalization}\\ \;\;\;+0.3\times S_{Reducedaccomplishment} \end{array}$$

A comprehensive score of <1.5 was mild, 1.5–3.5 was moderate and ≥3.5 indicated severe job burnout (Von Känel et al., 2016). These cutoff values are clinically relevant: those individuals with comprehensive scores of 1.5 or higher had significantly reduced physical and mental health compared to those with scores below 1.5. The Cronbach’s α values of job burnout and its three sub-dimensions in this study were 0.902, 0.899, 0.945, and 0.913, respectively.

Ability was derived from the academic performance records obtained from the interns’ colleges, and data-centric processing was used to avoid the collinearity problem. For attitude, according to the viewpoints of Consiglio et al. (2013) and Lee et al. (2018), a 5-item scale was revised from the usefulness scale, and 5-point Likert scoring was adopted, and 1 item was reverse-scored and reassigned during the analysis. After deleting the variables with a low factor loading, the Cronbach’s α value was 0.892. Self-efficacy was measured on the general self-efficacy scale revised by Wang et al. (2001); its Cronbach’s α was 0.843. Job satisfaction used a 5-item Likert-type scale compiled by Judge et al. (2001) for which higher score indicated greater job satisfaction. The Cronbach’s α value of job satisfaction in this study was 0.875. Perceived supervisor support and perceived co-worker support were based on the scale prepared by Settoon and Mossholder (2002) and Susskind et al. (2003), revised into a 6-item scale separately, two items of which were reverse-scored. According to the 5-point Likert-type scoring, the higher the score after reassignment, the stronger the sense of support. The Cronbach’s α values of support from supervisors and co-workers obtained after deleting two items with low loading were 0.913 and 0.888, respectively. Some non-Chinese scales were translated and revised in both directions to ensure the accuracy of the final Chinese scales. The reliability of all the variables was good.

Data on demographics and the nature of work included gender, hotel attributes, shift work, length of adaptation, internship allowance, workload, etc.

## Research results

### Descriptive statistics

The demographics and work characteristics of the sample are shown in Table 1. Males accounted for 29.5% and females accounted for 70.4%, and females who chose the hospitality industry accounted for the majority. In total, 56.2% of students worked as interns in international chain hotels, non-international hotels accounted for 43.8, and 81.27% of interns worked in five-star hotels. The interns who had experience with shift work accounted for 45.4, and 54.6% had never experienced this. Moreover, 94.2% of the interns became used to the job within 3 months, which shows that most students could change their roles soon after entering the hotel. In terms of the internship allowance, 45.4% of interns thought the allowance was significantly lower than expected, while 51.5% thought it was moderate. In total, 54.1% said that their work was heavy.

**Table 1:** The demographic and working characteristics of the sample.

Variables		Frequency (%)
Gender	Male	112 (29.6%)
	Female	267 (70.4%)
The attributes of the hotel	Non-international hotel	166 (43.8%)
	International hotel	213 (56.2%)
	Non-five-star hotel	71 (18.73%)
	Five-star hotel	308 (81.27%)
Shift work	Yes	172 (45.38%)
	No	207 (54.62%)
Length of adaptation	Less than 1 month	168 (44.33%)
	Less than 3 months and more than 1 month	189 (49.87%)
	More than 3 months	22 (5.80%)
Intern allowance	Falls short of expectations	172 (45.38%)
	Meets or exceeds expectations	207 (54.62%)
Workload	Heavy	205 (54.09%)
	Not heavy	174 (45.91%)

### Common method bias test

Because the data had been collected by the same source, in order to reduce the impact of the common method bias problem on the research results, this study adopted the Harman single factor test method to conduct a homologous variance test. That is, after all variables were put together for exploratory factor analysis, if there was only one factor to be parsed out, or if the first factor could explain most of the variation, this indicated that there was a common method bias. The results showed that there were seven factors with eigenvalues greater than 1, and the explanatory variation of the first factor was only 14.16%, which is far below the critical value of 40%. Therefore, it was judged that there was no serious common bias in the data of this study.

### Correlation analysis

SAS9.4 and Mplus7.4 were used for data analysis in this study. The results of the confirmatory factor analysis and correlation analysis are shown in Table 2. Under the condition that the convergent validity of each variable is guaranteed, the AVE square root of all variables was greater than their correlation coefficients with other variables, so the constituent variables of this model all had good discriminant validity.

**Table 2:** The result of the correlation analysis.

	ABI	SLE	ATT	JBS	PSS	PCS	EE	DP	PA	JBB
ABI	-									
SLE	0.020	0.863^a^								
ATT	0.064	0.539	0.861^a^							
JBS	0.029	0.603	0.584	0.767^a^						
PSS	−0.068	0.502	0.502	0.584	0.851^a^					
PCS	−0.035	0.517	0.561	0.564	0.683	0.816^a^				
EE	0.021	−0.395	−0.410	−0.526	−0.360	−0.370	0.810^a^			
DP	0.016	−0.418	−0.506	−0.592	−0.404	−0.416	0.786	0.902^a^		
PA	−0.155	−0.360	−0.349	−0.418	−0.298	−0.339	0.133	0.215	0.798^a^	
JBB	−0.043	−0.506	−0.548	−0.666	−0.459	−0.485	0.862	0.887	0.543	–

ABI, ability; SFE, self-efficacy; ATT, attitude; JBS, job satisfaction; PSS, perceived supervisor support; PCS, perceived co-worker support; EE, emotional exhaustion; DP, depersonalization; PA, reduced personal accomplishment; JBB, job Burnout.

a Square root of AVE.

### The status quo of job burnout of interns

As shown in Table 3, the comprehensive score of job burnout was 2.50±1.10 , the score of emotional exhaustion was 2.35±1.29 , the score of depersonalization was 2.18±1.58 and the score of reduced personal accomplishment was 3.01±1.54 . The emotional exhaustion and depersonalization of interns were at mild level. However, job burnout (on a scale of 1.5–3.5) and reduced personal accomplishment (on a scale of 3–5) were moderate, which H1 was supported. This is consistent with the research conclusions of Gavish and Friedman (2010), who found that job burnout at the initial stage of a career, including during an internship, should attract more attention from society and academia.

**Table 3:** The job burnout status of interns.

Variables	M	SD	Mild (%)	Moderate (%)	Severe (%)
Job burnout	2.50	1.10	67.3	22.1	12.4
Emotional exhaustion	2.35	1.29	66.2	18.5	15.5
Depersonalization	2.18	1.58	60.9	12.7	17.9
Reduced personal accomplishment	3.01	1.41	33.2	51.5	14.2

### Hierarchical multiple regression analysis

#### An analysis of the antecedents of job burnout

The results of the hierarchical multiple regression using SAS 9.4 are shown in Table 4. In Model 1, only the control variables were added. In this study, length of adaptation (0 = no more than 3 months, 1 = more than 3 months), workload (0 = not heavy, 1 = heavy) and shift work (0 = no, 1 = yes) was converted to dummy variable. The *F*-value of model regression was 15.99 (*p* < 0.0001), indicating that the overall effect of regression was very significant. The results showed that length of adaptation and workload were negatively correlated with burnout, while there was no significant correlation between shift work and burnout. In Model 2 and Model 3, in addition to the control variables, personal factors (ability, attitude and self-efficacy) and contextual factors (perceived supervisor support and perceived co-worker support, and job satisfaction) were added separately. The *F* value of M2 and M3 were 43.18 (*p* < 0.0001) and 58.80 (*p* < 0.0001), respectively. The regression effect of the two models was very significant.

**Table 4:** Analysis of the antecedents of job burnout.

Variables	Y = Job Burnout					
	M1		M2		M3	
	*β*	*t*	*β*	*t*	*β*	*t*
Length of adaptation	0.194^***^	3.97	0.124^***^	3.09	0.118^***^	3.15
Workload	0.260^***^	5.32	0.182^***^	4.50	0.112^***^	2.90
Shift work	0.063	1.29	0.013	0.32	0.002	0.06
Ability			−0.026	−0.65		
Attitude			−0.354^***^	−7.37		
Self-efficacy			−0.278^***^	−5.86		
PSS					−0.022	−0.41
PCS					−0.146^***^	−2.75
Job satisfaction					−0.529^***^	−10.86
*F*	15.99^***^		43.18^***^		58.80^***^	
Δ*R*^2^	0.106		0.401		0.479	

PSS, perceived superior support; PCS, perceived co-worker support.

*** *p* < 0.001.

The results showed that in terms of individual factors, attitude ( $\beta_{additude}=-0.354,t=-7.37$ ) and self-efficacy ( $\beta_{self-efficacy}=-0.278,t=-5.86$ ) had negative effects on job burnout. However, ability ( $\beta_{ability}=-0.026,t=-0.65$ ) had no statistically significant effect on job burnout. On the other hand, in terms of contextual factors, both perceived co-worker support ( $\beta_{perceived\;\mathrm{co}-\mathrm{worker}\;support}=-0.146,t=-2.75$ ) and job satisfaction ( $\beta_{job\;satisfaction}=-0.529,t=-10.86$ ) had significant negative impacts on job burnout. This is consistent with the results of most studies in the literature (Wu et al., 2009; Zhu et al., 2019). In contrast, perceived supervisor support ( $\beta_{perceivedsupervisorsupport}=-0.022,t=-0.41$ ) had no significant impact on job burnout, which is similar to the results of Duan and Xie (2015).

#### Analysis of the antecedents of the sub-dimensions of job burnout

Subsequently, we analyzed the antecedents of the sub-dimensions of job burnout, and the results are shown in Table 5. The significance level of obtained F value showed that the overall regression effect of all the constructed models was significant.

**Table 5:** Analysis of the antecedents of the sub-dimensions of job burnout.

Variables	Y = EE		Y = DP		Y = PA	
	*β*	*t*	*β*	*t*	*β*	*t*
Ability	0.025	0.57	0.021	0.49	−0.122^*^	−2.58
Attitude	−0.242^***^	−4.61	−0.358^***^	−7.06	−0.219^***^	−3.90
Self-efficacy	−0.216^***^	−4.18	−0.184^***^	−3.68	−0.249^***^	−4.49
PSS	−0.014	−0.23	−0.023	−0.40	−0.014	−0.21
PCS	−0.095	−1.59	−0.104	−1.81	−0.144^*^	−2.20
Job satisfaction	−0.395^***^	−7.17	−0.465^***^	−8.81	−0.366^***^	−6.07

EE, emotional exhaustion; DP, depersonalization; PA, reduced personal accomplishment; PSS, perceived superior support; PCS, perceived co-worker support.

* *p* < 0.05.

*** *p* < 0.001.

Self-efficacy ( $\beta_{self-efficacy}=-0.216,t=-4.18$ ), attitude ( $\beta_{attitude}=-0.242,t=-4.61$ ) and job satisfaction ( $\beta_{job\;satisfaction}=-0.395,t=-7.17$ ) had a negative correlation with emotional exhaustion. However, ability ( $\beta_{ability}=0.025,t=0.57$ ), perceived supervisor support ( $\beta_{perceivedsupervisorsupport}=-0.014,t=-0.23$ ), and perceived co-worker support ( $\beta_{perceived\;co-workersupport}=-0.095,t=-1.59$ ) had no statistically significant influence on emotional exhaustion.

Similarly, self-efficacy ( $\beta_{self-efficacy}=-0.184,t=-3.68$ ), attitude ( $\beta_{attitude}=-0.358,t=-7.06$ ) and job satisfaction ( $\beta_{job\;satisfaction}=-0.465,t=-8.81$ ) had a negative impact on depersonalization. However, ability ( $\beta_{ability}=0.021,t=0.49$ ), perceived supervisor support ( $\beta_{perceivedsupervisorsupport}=-0.023,t=-0.40$ ) and perceived co-worker support ( $\beta_{perceived\;co-workersupport}=-0.104,t=-1.81$ ) were not statistically correlated with depersonalization.

In addition, ability ( $\beta_{ability}=-0.122,t=-2.58$ ), self-efficacy ( $\beta_{self-efficacy}=-0.249,t=-4.49$ ), attitude ( $\beta_{attitude}=-0.219,t=-3.90$ ), job satisfaction ( $\beta_{job\;satisfaction}=-0.366,t=-6.07$ ) and perceived co-worker support ( $\beta_{perceived\;co-workersupport}=-0.144,t=-2.20$ ) had a negative impact on reduced personal accomplishment. In contrast, perceived supervisor support ( $\beta_{perceivedsupervisorsupport}=-0.014,t=-0.21$ ) had no statistically significant influence on reduced personal accomplishment.

In summary, hypothesis H2_2, H2_3, and H2_4 can be supported. However, part of H2_1 and H2_6 were supported and H2_5 was rejected.

## Conclusions and discussion

### Conclusion

Job burnout mostly occurs in the professional field where people are the objects of service. It refers to the mental state of an individual showing emotional exhaustion, depersonalization and reduced personal accomplishment. Job burnout can induce deterioration of employees’ health or poor performance, and it is related to negative personal and organizational performance. For more than 50 years, scholars (e.g., Li and Shi, 2003; Hsieh et al., 2021 etc.) have carried out unremitting research on the antecedents and consequences of job burnout in many fields.

Due to the bias that burnout is caused by accumulated stress over a long period of time, burnout in the early stage of a career has received less attention from scholars. On the contrary, Gavish and Friedman (2010) argued that burnout, although a gradual process, is already budding in pre-employment training or internships. Early in the career, especially during the internship phase of transition from students to employees, interns have to accept new roles and tasks that are completely different from school life. In addition, they must adapt to the new environment as soon as possible, integrate into a new team and transition from theory to practice. The imbalance between the high standards and strict requirements of the internship unit and the students’ own abilities can easily cause them to burn out. However, few scholars have studied job burnout during this period. Therefore, this study took hotel interns as the object and empirically studied the level and antecedents of job burnout and antecedents of job burnout’s sub-dimensions, which will help higher vocational colleges to better organize and implement internships, minimize the job burnout of interns and lay a theoretical foundation for hotels to recruit energetic future employees.

This study first examined the level of job burnout based on the MBI-GS scale in Chinese characteristics modified by Chinese scholar Li and Shi. The results that according to Von Känel et al. (2016)'s original Burnout Risk Index (BRIX-O) formula showed that the job burnout of interns was at a moderate level. In the investigation of the sub-dimensions, emotional exhaustion and depersonalization were mild, but reduced personal accomplishment was moderate. It can be seen that in the process of applying theory to practice, students are suffering from reduced personal accomplishment due to unskilled work tasks, etc. This result is slightly different from that of Kokkinos and Stavropoulos (2014), who took student teachers as the research objects. Hospitality industry interns have developed preliminary job burnout.

Secondly, based on the transaction model, this study attributed the antecedents of job burnout into two categories: personal factors and contextual factors. The results showed that personal factors such as attitude and self-efficacy, and contextual factors such as perceived co-worker support and job satisfaction all had a negative impact on job burnout, which is consistent with numerous research results (e.g., Wu et al., 2009). That is, if interns have a positive attitude towards their internship or show stronger perseverance despite being in a stressful situation, their level of job burnout will be lower. Similarly, interns with high job satisfaction or with more support from colleagues at work had a low rate of burnout. However, the influence of ability and perceived supervisor support on job burnout was not significant.

Thirdly, this study examined the influencing factors of each sub-dimension of job burnout. This study found that self-efficacy, attitude and job satisfaction all had a negative impact on the three sub-dimensions of job burnout. Ability and perceived co-worker support only had a negative impact on reduced personal accomplishment. There was no statistical correlation between perceived supervisor support and the three sub-dimensions. The impact of ability was relatively weak, and follow-up investigations have found that this may be related to the COVID-19 pandemic. At the beginning of 2020, the sudden COVID-19 outbreak blocked access to school for the interns for study and practical training. Some students had not undergone practical training before the internship. Only a few students received hasty short-term training and were not fully prepared for the internship. Therefore, the role of ability needs further research and discussion. Also, interns are not regular employees of the hotel and may be less emotionally invested in by the supervisors or colleagues. In particular, support from supervisors is generally based on constructive opinions. Interns may not clearly feel support from their supervisors.

### Theoretical contribution and implications for management

First, the existing literature is affected by the conclusion that “burnout is caused by long-term accumulation,” and few scholars have researched job burnout in the early stages in the workplace. One of the theoretical contributions of this study is to take the lead in exploring the level and causes of job burnout in the initial stages of a career. The results confirm that the internship stage is the budding phase of the gradual process of hotel employees’ job burnout. Secondly, this study breaks the convention of job burnout studies starting from a single perspective of personal factors or contextual factors, and integrates the two based on the transaction model to comprehensively demonstrate the antecedents of job burnout. Third, this study not only examines the level and antecedents of burnout, but also empirically demonstrates the influencing factors of the three sub-dimensions of job burnout, so the study’s conclusion is more detailed.

From the conclusions of this study, it can be seen that the job burnout of interns is affected by personal and contextual factors, and requires benign interventions from hotels and higher vocational colleges to actively construct an environment that can alleviate burnout.

On the personal side, interns should cultivate a good mentality and a positive attitude, face up to the objective existence of stressors in the process of role change, improve their own psychological adjustment levels, enhance their self-confidence, dare to face challenges and respond correctly. During the internship, the interns should alternate work with rest, and actively seek effective strategies and ways to solve problems, or receive emotional counseling. Interns should practice running or other physical activities to induce endorphins, help their focus, improve their mind and effectively relieve stress.

On the corporate side, in addition to economic returns, hotels should treat interns with respect and give them equal treatment, consider them to be members of the company, create a harmonious working atmosphere and enhance their sense of belonging and honor. In particular, supervisors, in addition to policy support, should delegate powers to improve the autonomy of interns, which is believed to alleviate the work pressure of novices. A good example is the Ritz-Carlton system, which grants $2000 to all employees, including interns, to write a Wow Story. In addition, supervisors should strive to improve the emotional-based management model, create a warm working environment and enhance the interns’ sense of internal support. On the other hand, according to the survey, hotel colleagues and interns are together day and night, so while guiding the business, they should also inject emotions to more effectively reduce the level of burnout in interns.

As for schools, various mechanisms should be established first to build a communication platform for interns. For example, some higher vocational colleges have implemented a corporate tutor system. The original intention was to formulate career plans for students and expand employment channels, but a good system cannot be a mere formality. Schools should refine the relevant policies, for example, preferentially giving priority to students who have visited their tutors, and directing these students to the department where their tutors work for internships. In other words, moving students from a familiar schoolteacher to a familiar mentor would reduce the strangeness of students taking their first steps in the workplace. We believe that the tutoring system will have good results in reducing job burnout. Secondly, higher vocational colleges should improve the class teaching quality in view of the high rate of hotel interns reporting reduced personal accomplishment. Classes should fully stimulate the enthusiasm of students to use their hands and brains, so that they can master theoretical knowledge and improve their professional skills related to digital operations such as PMS, POS, etc., which are necessary for internships and jobs in the modern hospitality industry, and thus improve their business proficiency in order to reduce the reduced personal accomplishment of interns.

### Limitations and future research

The survey objects of this study were limited to hotel management interns in higher vocational colleges, and the cross-section was relatively narrow. The data cross-section will be broadened in the future to analyze the differences between college students and undergraduates in terms of job burnout.

## Data availability statement

The raw data supporting the conclusions of this article will be made available by the authors, without undue reservation.

## Ethics statement

The studies involving human participants were reviewed and approved by Chungnam National University. The patients/participants provided their written informed consent to participate in this study.

## Author contributions

All authors listed have made a substantial, direct, and intellectual contribution to the work, and approved it for publication.

## Funding

This research is supported by following programs: The Ministry of Education of the Republic of Korea and the National Research Foundation of Korea (NRF-2021S1A5B8096365), Horizontal project of Chinese Dictionary Research Center of China (CSZX-YB-202012). Shandong Humanities and Social Sciences Project (2022-ZXWL-14).

## Conflict of interest

The authors declare that the research was conducted in the absence of any commercial or financial relationships that could be construed as a potential conflict of interest.

## Publisher’s note

All claims expressed in this article are solely those of the authors and do not necessarily represent those of their affiliated organizations, or those of the publisher, the editors and the reviewers. Any product that may be evaluated in this article, or claim that may be made by its manufacturer, is not guaranteed or endorsed by the publisher.

## References

[ref1] Ahola K. Honkonen T. Isometsä E. Kalimo R. Nykyri E. Aromaa A.. (2005). The relationship between job-related burnout and depressive disorders–results from the Finnish health 2000 study. J. Affect. Disord. 88, 55–62. doi: 10.1016/j.jad.2005.06.004, PMID: 16038984

[ref2] Aloe A. M. Amo L. C. Shanahan M. E. (2014). Classroom management self-efficacy and burnout: a multivariate meta-analysis. Educ. Psychol. Rev. 26, 101–126. doi: 10.1007/s10648-013-9244-0

[ref3] Blomberg S. Rosander M. (2020). Exposure to bullying behaviours and support from co-workers and supervisors: a three-way interaction and the effect on health and well-being. Int. Arch. Occup. Environ. Health 93, 479–490. doi: 10.1007/s00420-019-01503-7, PMID: 31828422 PMC7118028

[ref4] Burger K. Samuel R. (2017). The role of perceived stress and self-efficacy in young people’s life satisfaction: a longitudinal study. J. Youth Adolesc. 46, 78–90. doi: 10.1007/s10964-016-0608-x, PMID: 27812840

[ref5] Cano-García F. Padilla-Munoz E. Carraso-Ortiz M. (2005). Personality and contextual variables in teacher burnout. Pers. Individ. Differ. 38, 929–940. doi: 10.1016/j.paid.2004.06.018, PMID: 26159952

[ref6] Chan D. W. (2003). Hardiness and its role in the stress-burnout relationship among prospective Chinese teachers in Hong Kong. Teach. Teach. Educ. 19, 381–395. doi: 10.1016/S0742-051X(03)00023-4

[ref7] Chan S. H. Wan Y. K. P. Kuok O. M. (2015). Relationships among burnout, job satisfaction and turnover of casino employees in Macau. J. Hosp. Mark. Manag. 24, 345–374. doi: 10.1080/19368623.2014.911712

[ref8] Chen H. Liu F. Pang L. Liu F. Fang T. Wen Y.. (2020). Are you tired of working amid the pandemic? The role of professional identity and job satisfaction against job burnout. Int. J. Environ. Res. Public Health 17, 1–14. doi: 10.3390/ijerph17249188, PMID: 33316964 PMC7764790

[ref9] Consiglio C. Borgogni L. Alessandri G. Schaufeli W. B. (2013). Does self-efficacy matter for burnout and sickness absenteeism? The mediating role of demands and resources at the individual and team levels. Work Stress 27, 22–42. doi: 10.1080/02678373.2013.769325

[ref10] Du H. J. Li Z. X. Liang S. S. Wang B. W. Xu F. G. (2021). Analysis of the impact of demographic characteristics of hospital managers on job burnout. Chin. Hosp. Manag. 41:93.

[ref11] Duan H. M. Xie Y. H. (2015). The statistical and test of social support on the relationship between work pressure and work attitude. Stat. Decis. 15, 94–97. doi: 10.13546/j.cnki.tjyjc.2015.15.026

[ref12] Gavish B. Friedman I. A. (2010). Novice teachers’ experience of teaching: a dynamic aspect of burnout. Soc. Psychol. Educ. 13, 141–167. doi: 10.1007/s11218-009-9108-0

[ref13] Goddard R. Goddard M. (2006). Beginning teacher burnout in Queensland schools: associations with serious intentions to leave. Aust. Educ. Res. 33, 61–75. doi: 10.1007/BF03216834

[ref14] Harjanti D. Todani F. A. (2019). Job burnout and employee performance in hospitality industry: the role of social capital. J. Teknik Ind. 21, 15–24. doi: 10.9744/jti.21.1.15-24

[ref15] He X. Y. (2011). The relationship between student burnout and academic achievement in local Normal universities. Mod. Educ. Manag. 7, 72–74. doi: 10.16697/j.cnki.xdjygl.2011.01.022

[ref16] Hoyer W. D. MacInnis J. D. (2013). Consumer behavior, 5th ed.; South Western College. Cengage Learning (Mason OH).

[ref17] Hsieh H. F. Liu Y. Hsu H. T. Ma S. C. Wang H. H. Ko C. H. (2021). Relations between stress and depressive symptoms in psychiatric nurses: the mediating effects of sleep quality and occupational burnout. Int. J. Environ. Res. Public Health 18:7327. doi: 10.3390/ijerph18147327, PMID: 34299778 PMC8303432

[ref18] Hu H. H. Cheng C. W. (2010). Job stress, coping strategies, and burnout among hotel industry supervisors in Taiwan. Int. J. Hum. Resour. Manag. 21, 1337–1350. doi: 10.1080/09585192.2010.483867

[ref19] Huang S. Y. B. Fei Y. M. Lee Y. S. (2021). Predicting job burnout and its antecedents: evidence from financial information technology firms. Sustainability 13:4680. doi: 10.3390/su13094680

[ref20] Judge T. A. Thoresen C. J. Bono J. E. Patton G. K. (2001). The job satisfaction-job performance relationship: a qualitative and quantitative review. Psychol. Bull. 127, 376–407. doi: 10.1037/0033-2909.127.3.376, PMID: 11393302

[ref21] Kalimo R. Pahkin K. Mutanen P. Topipinen-Tanner S. (2003). Staying well or burning out at work: work characteristics and personal resources as long-term predictors. Work Stress 17, 109–122. doi: 10.1080/0267837031000149919, PMID: 36311615

[ref22] Kilroy S. Bosak J. Chênevert D. Flood P. C. Hill K. (2021). Reducing burnout among nurses: the role of high-involvement work practices and colleague support. Health Care Manag. Rev. 47, 115–124. doi: 10.1097/HMR.0000000000000304 33428348

[ref23] Kokkinos C. M. Stavropoulos G. (2014). Burning out during the practicum: the case of teacher trainees. Educ. Psychol. 36, 548–568. doi: 10.1080/01443410.2014.955461

[ref24] Lee S. Baumgartner H. Winterich K. P. (2018). Did they earn it? Observing unearned luxury consumption decreases brand attitude when observers value fairness. J. Consum. Psychol. 28, 412–436. doi: 10.1002/jcpy.1028

[ref25] Li Z. F. Chen Q. (2012). Research on the affecting factors of job burnout for travel agency managers. Tour. Trib. 27, 92–100.

[ref26] Li C. P. Shi K. (2003). The influence of distributive justice and procedural justice on burnout. Acta Psychol. Sin. 35, 677–684.

[ref27] Liu T. Chen X. M. Lu X. R. Yang Y. (2021). The influence of positive parenting style on coping style of middle school students: the mediating role of social support and self-efficacy. Stud. Psychol. Behav. 19, 507–514.

[ref28] Lizano E. L. Mor Barak M. (2015). Job burnout and affective wellbeing: a longitudinal study of burnout and job satisfaction among public child welfare workers. Child Youth Serv. Rev. 55, 18–28. doi: 10.1016/j.childyouth.2015.05.005

[ref29] Lu A. C. C. Gursoy D. (2016). Impact of job burnout on satisfaction and turnover intention: do generational differences matter? J. Hosp. Tour. Res. 40, 210–235. doi: 10.1177/1096348013495696

[ref30] Lu M. H. Luo J. Chen W. Wang M. C. (2021). The influence of job satisfaction on the relationship between professional identity and burnout: a study of student teachers in Western China. Curr. Psychol. 41, 289–297. doi: 10.1007/s12144-019-00565-7

[ref31] McAllister C. P. Harris J. N. Hochwarter W. A. Perrewé P. L. Ferris G. R. (2017). Got resources? A multi-sample constructive replication of perceived resource Availability’s role in work passion-job outcomes relationships. J. Bus. Psychol. 32, 147–164. doi: 10.1007/s10869-016-9441-1

[ref32] Pinna R. Simone S. D. Cicotto G. Malik A. (2020). Beyond organisational support: exploring the supportive role of co-workers and supervisors in a multi-actor service ecosystem. J. Bus. Res. 121, 524–534. doi: 10.1016/j.jbusres.2020.02.022

[ref33] Prentice C. Thaichon P. (2019). Revisiting the job performance: burnout relationship. J. Hosp. Mark. Manag. 28, 807–832. doi: 10.1080/19368623.2019.1568340

[ref34] Schaufeli W. B. Bakker A. B. (2004). Job demands, job resources, and their relationship with burnout and engagement: a multi-sample study. J. Organ. Behav. 25, 293–315. doi: 10.1002/job.248

[ref35] Settoon R. P. Mossholder K. W. (2002). Relationship quality and relationship context as antecedents of person-and task-focused interpersonal citizenship behavior. J. Appl. Psychol. 87, 255–267. doi: 10.1037/0021-9010.87.2.255, PMID: 12002954

[ref36] Susskind A. M. Kacmar K. M. Borchgrevink C. P. (2003). Customer service providers’ attitudes relating to customer service and customer satisfaction in the customer-server exchange. J. Appl. Psychol. 88, 179–187. doi: 10.1037/0021-9010.88.1.179, PMID: 12675405

[ref37] Taylor M. McLean L. Bryce C. I. Abry T. Granger K. L. (2019). The influence of multiple life stressors during teacher training on burnout and career optimism in the first year of teaching. Teach. Teach. Educ. 86:102910. doi: 10.1016/j.tate.2019.102910

[ref38] Tian J. Mao Y. Q. Xiong H. X. (2021). The effect of transformational leadership on teachers’ job burnout: the chain mediating role of social emotional competence and well-being. Psychol. Dev. Educ. 37, 743–751. doi: 10.16187/j.cnki.issn1001-4918.2021.05.16

[ref39] Tu Y. Zhang S. (2015). Loneliness and subjective well-being among Chinese undergraduates: the mediating role of self-efficacy. Soc. Indic. Res. 124, 963–980. doi: 10.1007/s11205-014-0809-1

[ref40] Von Känel R. Van Nuffel M. Fuchs W. J. (2016). Risk assessment for job burnout with a Mobile health web application using questionnaire data: a proof of concept study. BioPsychoSocial Med. 10, 1–13. doi: 10.1186/s13030-016-0082-4 PMC5093935 27822296

[ref41] Wang C. K. Hu Z. F. Liu Y. (2001). Research on the reliability and validity of general self-efficacy scale. Chin. J. Appl. Psychol. 7, 37–40.

[ref42] Wei X. M. He J. Zhao W. D. Peng L. P. (2021). The relationship between transition shock and job burnout of standardized training nurses in Sichuan Province. Med. Soc. 34:88. doi: 10.13723/j.yxysh.2021.07.016

[ref43] Wu X. Chi J. M. He X. F. (2009). Research on the job satisfaction and job burnout of research-type university teachers. Mod. Educ. Manag. 7, 57–60. doi: 10.16697/j.cnki.xdjygl.2009.07.019

[ref44] Yan Z. Mansor Z. D. Choo W. C. Abdullah A. R. (2021). Mitigating effect of psychological capital on employees’ withdrawal behavior in the presence of job attitudes: evidence from five-star Hotels in Malaysia. Front. Psychol. 12:617023. doi: 10.3389/fpsyg.2021.617023, PMID: 33868086 PMC8044991

[ref45] Yetgin D. Benligiray S. (2019). The effect of economic anxiety and occupational burnout levels of tour guides on their occupational commitment. Asia Pac. J. Tour. Res. 24, 1–16. doi: 10.1080/10941665.2018.1564681

[ref46] Yu X. Wang P. Zhai X. Dai H. Yang Q. (2015). The effect of work stress on job burnout among teachers: the mediating role of self-efficacy. Soc. Indic. Res. 122, 701–708. doi: 10.1007/s11205-014-0716-5, PMID: 35145457

[ref47] Zhai Q. Lindorff M. Cooper B. (2013). Workplace Guanxi: its dispositional antecedents and mediating role in the affectivity-job satisfaction relationship. J. Bus. Ethics 117, 541–551. doi: 10.1007/s10551-012-1544-7

[ref48] Zhu J. N. Y. Lam L. W. Lai J. Y. M. (2019). Returning good for evil: a study of customer incivility and extra-role customer service. Int. J. Hosp. Manag. 81, 65–72. doi: 10.1016/j.ijhm.2019.03.004

